# Remediating Cambridge: Human and Horse Co-Relationality in a Culture of Mis-Re-Presentation

**DOI:** 10.3390/ani15020194

**Published:** 2025-01-13

**Authors:** Francesca A. Brady, Jennifer McDonell

**Affiliations:** English Literary Studies, School of Humanities, Arts and Social Sciences (HASS), University of New England, Armidale, NSW 2351, Australia

**Keywords:** Cambridge, laminitis, media misrepresentation, animal agency, agencement, popular media, feminist care tradition in animal ethics, intersectional analysis, Australian Equestrian cultures, autoethnography, phenomenology, equine welfare, innovative behaviour in equines

## Abstract

This case study aims to problematise concepts of equine and human co-relational agency in the context of ‘mis-re-presentations’ in the Australian media of harms experienced by the Anglo Arab stallion, Cambridge, following his development of laminitis and his consequent confinement at a leading national Equestrian centre. Autoethnographic narrative is used to retrospectively and selectively narrate the evolving relationship between Cambridge and his owners, farrier, and treating veterinarians within the dominant housing and veterinary practices and welfare paradigms in equestrian culture of 1990’s Australia. Structured author/owner autoethnographic vignettes are framed by newspaper and internet reportage to highlight a productive tension between the public mediation of the case, and what it means to be fully embodied in relationship with an equine companion agent within a particular, racialised, gendered, and biopoliticised location. Adopting a phenomenologically informed intersectional feminist ethics of care perspective, a counternarrative to the gendered, racialised and essentialising rights-based judgements about Cambridge’s illness and eventual death that dominated the popular media is provided. Crucially, the autoethnographic vignettes are chosen to capture the corporeal reciprocity and rapport of forces that produced a co-created agentivity that characterised the horse’s birth, training, and treatment. The embodied interspecies knowledge that informs the training and care of equines (and all animal species) is always historically situated within permeable, dynamic worlds of self and other that are fluid, contextual, and always in relation. It is suggested that the case of Cambridge illustrates how competing stakeholder investments in animal welfare can play out in the public mediation of particular cases in ways that exclude their historical and interspecies situatedness and serve to reinforce dominant ideologies governing human and animal relationships.

## Simple Summary

The medical treatment of Cambridge, an Anglo-Arab stallion, was misrepresented in the Australian media. His carers were trolled on the Cyberhorse website in Victoria, Australia, in the 1990s. Emotive misrepresentations, some anonymous, led to public misogynistic and racist labelling of his owners as ‘wogs’ and witches. Other misrepresentations maligned the reputation of the vets and farrier (blacksmith) who treated Cambridge and led the Royal Society for the Prevention of Cruelty to Animals (RSPCA) to publicly doubt the expertise of the practicing equine veterinarian and consulting specialist. Real-world harm was caused and claims based on media representations were investigated by the Veterinary Practitioners Registration Board of Victoria. The Victorian Supreme Court passed an injunction to restrain the Executive Officer, or any RSPCA inspector, from ‘killing, touching, or otherwise interfering’ with Cambridge. Another writ was obtained preventing the Werribee Park Equestrian Centre (WPEC) from forcibly moving Cambridge or his companion horses while Cambridge was undergoing veterinary treatment for laminitis. The Centre stopped advising Cambridge’s carers of changes to his environment that would adversely affect him, yet transporting Cambridge elsewhere would have been a death sentence. Media reportage of Cambridge’s welfare and treatment failed to consider the co-relationality that distinguished his treatment, or accord the horse agency in this process. Throughout, Cambridge proved an exceptional horse and patient, exhibiting what appeared to be agential adaptations to circumstances and his overall health and wellbeing. Cambridge’s story urges us to consider that when making decisions about the lives of animals we should not make decisions for them, but *with* them.

## 1. Introduction

This case study adopts a phenomenologically informed intersectional ethics of care perspective to present a counternarrative to ‘re-presentations’ in the Australian media of harms experienced by the Anglo Arab stallion, Cambridge while housed at Werribee Park Equestrian Centre-in Victoria from 1996 to 2002. The emotive, polarised, human-centred media storm surrounding Cambridge’s protracted illness and housing following his development of laminitis—colloquially termed founder—leveraged legitimate concern by the Australian public about the welfare of elite sport horses. However, media reportage was also characterised by gendered and racialised discourses that drew on metaphors and tropes adapted to animalising humans, reminding us that gender and species stereotypes continuously reinvigorate each other in a co-joined logic of subordination to assert a universalist narrative about the moral consideration of animal suffering. More broadly, this case study raises important questions about the media’s role in responsibly representing equine welfare. Structured author/owner autoethnographic vignettes are used to highlight a productive tension between the public mediation of the case, and the affective dimension of the horse-human relationship. Specifically, this case study aims to use autoethnographic narrative to show what it means to be fully embodied in relationship with an equine companion agent within a particular, racialised, gendered, and biopoliticised location. The overdetermined media stereotyping of the horse and his owners and carers, further serves to underline the fundamental instability of the human and animal divide that allows for the animalisation of women and other subaltern groups and conversely, the anthropmorhising or humanisation of animals in human language systems.

After firstly explaining the methodology underpinning the case study, examples of the media misrepresentations made against Cambridge, his owners and veterinarians are cited. These representations motivated the RSPCA to publicly call for Cambridge’s euthanasia, noting that these representations form part of the publicly available records of the case. A counter narrative is offered using author/owner autoethnographic vignettes to highlight biographical episodes in Cambridge’s life beginning with his birth. The narrative also describes early attempts at ‘breaking-in’ Cambridge, which he resisted, followed by the implementation of an entirely new training regimen, onset of laminitis, the disease’s causation and consequences for Cambridge, and the protocols initiated by the treatment team to ensure his welfare. Importantly, the vignettes aim to illustrate the agential rapport between Cambridge and his owner, veterinarians and farrier, which both guided and enabled his treatment for the disease, particularly what seemed to be the horse’s attempts to alter species-specific innate behaviour as he tried to adjust to his disabled condition. This narration concludes with an appeal to contemporary theorisation of human-animal relationships, particularly Donna Haraway’s conceptualisation of ‘situated knowledges’, Vinciane Despret’s explication of ‘agencement’ and the feminist care tradition in animal ethics, as pioneered by Josephine Donovan and Carol J. Adams. The feminist care standpoint advocates a shift away from epistemological and ulitilitarian approaches to ethical deliberation to include feminist witnessing of animal suffering, which in the case of the horse-human relationship might be experienced as an expression of power *with* the horse, rather than coercive, objectifying power *over* structures imposed upon the horse, the latter drawing legitimacy from unquestioned anthropocentric assumptions about human exceptionalism.

The autoethnographic vignettes provided herein have been crafted to capture the corporeal reciprocity and rapport of forces that produced a co-created agentivity that was observed by various human actors in Cambridge’s life to have characterised his birth, treatment and eventual death. The phenomenological perspective that informs the autobiographical vignettes stands in stark contrast to the often wildly speculative representations of the horse’s incarceration and veterinary treatment on internet sites and in the Australian press. Phenomenological enquiry asks that we foreground the experiential in our writing and equitation practices alike. It requires that we ‘re-learn to look at the world as we meet it’ [1] (p. 184), then and there, through a ‘kind of attention and wonder, a demand for awareness, a will to grasp the sense of the world in its nascent state’ [2] (p. 22). In short, it asks us to recognise the corporeality of intersubjectivity. While not automatically phenomenological, as an approach to qualitative research and writing that is anchored in reflexive praxis, autoethnography is a productive phenomenological resource [3,4]. In this case study, autoethnography ‘articulates the intersection of cognitive and somatic experience’ [5], through its ‘centering of the author, [which] allows intimate aspects of understandings and experiences, often inaccessible to researchers, to become a part of narratives and contribute to the field’ [6] (p. 12). Autoethnography ‘balances intellectual and methodological rigor, emotion, and creativity’ [7] (p. 2) to ‘offer nuanced, complex, and specific knowledge about particular lives, experiences, and relationships rather than general information about large groups of people’ [7] (p. 21). Arthur Bochner states that autoethnography ‘usually depicts people struggling with problematic circumstances…autoethnographies show people in the process of figuring out what to do, how to live, and what their struggles mean’ [8]. This case study narrates how Cambridge’s owner, veterinarians and farrier navigated his treatment for laminitis, remaining attentive to the problematic situatedness of the horse’s circumstances and fully engaged in an ongoing process of adjustments and discovery. Autoethnography also authorises, *inter alia*, the use of thick description to critically analyse personal experience in order to understand cultural experience [9]. In doing so it ‘often make[s] use of conventions of creative writing’, including use of the first-person voice, as evident in the autoethnographic vignettes included in this case study [5]. However, it is conceded that first-person narrativity is inherently anthropocentric and ‘othering’ because by necessity it ‘projects the structures of human subjectivity on animals’, and thereby necessitates an ‘acknowledgement as to the limits of interspecies empathy’ [10] (p. 14).

Whilst recognising the limitations that haunt humancentric methodologies, the literary techniques used in this paper are tentatively directed towards promoting ethical attentiveness and sympathy in our relationships with animals, and recognising a link between popular mediations of animal welfare discourses and the continuing subjugation of women and the human domination of animals. Seeking to reclaim feminine embodiment and emotionality as the foundation for feminist animal care ethics, Josephine Donovan and Carol J. Adams call for a ‘unified radical and cultural feminist approach to animal issues, repositioning the ethic of care within the political perspective of the radical feminist tradition’ [11] (p. 10). Drawing parallels between violence against women and violence against animals, feminist animal care theory draws on the feminist care theory tradition to value relationality (rather than autonomy or individualism) and ‘emotions such as empathy as important modes of knowledge’ [12] (p. 4). An operative assumption in this tradition is that the oppression of women and animals is a structurally interrelated feature of an ableist, racist, sexist, speciesist violent hierarchy that privileges human, able-bodied, white, heterosexual masculinity as compulsively normative. In Donovan’s words, ‘We need … to reorient or reemphasize that care theory means listening to other life-forms regardless of how alien they may seem to us and incorporating their communications into our moral reaction to them’ [13] (p. 315). 

While women are more likely to support animal rights than men, and historically the animal advocacy movement has been disproportionately initiated and led by women, particularly in the United Kingdom, it is important to caution against making automatic equivalences between women and ethical decision making in relation to animal welfare [14,15,16]. Recent studies on moral reasoning and action choices in both animal and human scenarios suggest that such choices relating to animal ethics issues are influenced by a range of factors, including demography, cultural and educational background and knowledge. Joy M. Verrinder and Clive J.C. Phillips, for example, have demonstrated that assessment tools such as the VetDIT can assist in understanding the relationship between intuitive action choices and moral reasoning in animal ethics decision-making [17]. While the approach adopted in this paper is not empirically based, we are mindful of the importance of perspective and privilege in the activity of moral theorising, and do not wish to impugn those actors who by virtue of their training and relationship to the horse, may have had alternative viewpoints. Rather, by foregrounding the phenomenological dimension of one horse-human relationship this paper aims to historicise care ethics as a situated practice in place and time and construes care as the symbolic rather than the actual voice of women [18], noting the potential of care as a gender-neutral activity. For example, the male veterinarians and farrier who treated Cambridge also exhibited ‘care’, both personally and professionally. To resolve the possible tension between narrow conceptions of femininity and feminism, the critique of media representations of Cambridge and his owners and carers is informed by an intersectional approach to the question of whose femininity is being discussed. In this vein, feminist commentators on the care tradition in animal ethics have called for a more critically informed understanding of care and a greater focus on animal agency [19,20,21], important enabling and qualifying emphases which are illustrated in the vignettes that form the core of this paper.

The autoethnographic vignettes, and accompanying discussion, also incorporate an element of feminist witnessing, which is also aligned to the feminist care tradition in animal ethics and involves ‘a moment of recognition of non-human animals in ways that draw our attention to the power imbalance between human and nonhuman animals and require us to rethink our multispecies lifeworlds’ [21] (p. 21) (also [22,23]). As such, the autoethnographic vignettes aim to capture moments of being physically, mentally, and emotionally present; practicing empathy; seeking to understand the complex and contradictory relationships of care, surveillance, and violence that impacted Cambridge’s truncated life, with a view to transforming these relationships to promote a sense of curiosity and humility in our communication with other animals. For example, Cambridge’s eruption of feral agency in resisting the human domination of breaking in, as well as other coercive practices associated with traditional and behaviourist learning regimes of equine training and management, in which he was subjected to power *over*. Conversely, restorative training and treatment regimes, founded upon a conception of equine agency as an assemblage of actions based on an interspecies reciprocity that produces agentivity are also described. We borrow Vinciane Despret’s theorisation of agencement [24] (pp. 29–44) to suggest that the subjective notion of autonomous intention or sympathetic projection that informs conventional notions of agency or horse ‘point of view’ are inadequate as an analytical frame through which to understand Cambridge’s responses to his protracted veterinary treatment during which he was stabled in housing conditions (from which he was unable to be removed due to detrimental aspects of transportation which would have worsened his condition) that impacted his welfare. By contrast, the conventional overdetermined conceptions of agency as encompassing freewill and moral responsibility, as rational, intentional and premediated, appeared to inform representations of Cambridge as a powerless victim of deliberative and protracted human abuse on popular equine internet sites and in national newspaper outlets by media personalities, external industry experts and animal welfare organisations who were unconnected to his treatment and eventual death. The question for this analysis is not whether or not horses have agency, but what ideological and representational work is performed by the imputation or denial of agency, human or non-human.

## 2. Re-Presenting Cambridge: Narrative and Counter Narrative

In May 2002, a post appeared on an equine industry internet site claiming that five years earlier at the Werribee Park National Equestrian Centre (WPEC) in Victoria, a stallion was found locked in a darkened box, and that to the contributor’s ‘horror’ the horse was still there [25] (p. 242). In a newspaper article titled “Dark Horses Not in Running” the claim was further pursued: ‘this sad and strange situation first became public palaver on a [now defunct] internet site called Cyberhorse, an online gossip spot for the equestrian and racing set. Two weeks ago, someone posted that three stallions were being kept in their stalls, in darkness, day after day and that they’d been locked up that way for some years’ [26].

These claims related to Cambridge, an Anglo Arab stallion bred, owned, trained, and eventually nursed by sisters Francesca and Allison Raffaele (Francesca Brady, née Raffaele, is co-author of this article. Allison, while part of Cambridge’s training, care and treatment, will only be nominally mentioned to respect her privacy). The other two stallions referred to were Cambridge’s sons, Copenhagen and Congressman. Often unmoderated, the internet site’s content amplified stereotyping of Cambridge, Copenhagen, and Congressman as hapless, neglected victims of financial interests, while vilifying contributors who questioned the legitimacy of such representations. Media reports on the living conditions of the horses inevitably extended to vilification of their owners along gender, class and racial lines: that the owners were women, had foreign surnames, and were “backstage” actors in the Australasian elite sport horse hierarchy, made them easy targets. A report by John Elder in Melbourne’s *The Age* newspaper stated: ‘You always hear them called “the sisters” instead of their names Francesca and Allison Raffaele, and you always hear the sisters described as “so secretive”. So when a bit of news leaks out, everybody knows about it’ [26]. Thousands of postings appeared on Cyberhorse [27] in the ensuing weeks. A protest group rapidly formed to lobby news and current affairs television programs, as well as morning, daytime, and evening talkback radio hosts, and Victorian newspaper outlets. Based on the content in the online forum and news media, the protest group lobbied the Bureau of Animal Welfare (BAW), and the Royal Society for the Prevention of Cruelty to Animals (RSPCA) to prosecute the owners for animal cruelty. They engaged a law firm to press for the euthanasia of Cambridge, and the removal of Copenhagen and Congressman into the care of a third party [28]: ‘We will get them—today’s meeting with the minister will be to get the other 2 horses seized and the Raffaeles prosecuted at every level we can find’ [25] (p. 329). Politicians, industry representatives, and radio personalities collaborated to draw public attention to the stallions’ supposed plight. The BAW communicated with, and the RSPCA acted upon representations made on Cyberhorse, despite the claims made being often vitriolic.

The hyperbolic use of such epithets as “dark”, “cold”, “darkened”, “plight”, “horror” and “cruel” proved to be emotive triggers for readers, appearing in almost every media report on all platforms. The gothic connotations of the language evoked images of the captive stallions being imprisoned for years on end in cold, comfortless, and darkened boxes by cruel, neglectful masters, much like the virtuous heroine typically incarcerated in a castle and pursued by a sadistic aristocrat in gothic novels, generating considerable visceral shock value. The ‘sisters’ who were second-generation Australians, were ethnically vilified based solely on their surnames: ‘It is amazing how often people of ethnic origin always feature in crimes of violence … and cruelty to animals … you show yourselves to be what you are—a very unpleasant, vicious, money hungry wog …’ [25] (p. 263). While newspaper reports branded Allison and Francesca as ‘the sisters’, and one going further to state that ‘the sisters have been living in the stables with them [the horses] performing “magic”’ [26], contributors on Cyberhorse showed no restraint. Another post was titled, ‘The Ugly Sisters’ evoking misogynistic stereotypes of Cinderella’s cruel stepsisters. A thread on Cyberhorse began:

*hail the evil ones—the Raphael [sic] sisters. Spit on them because they are witches …search their bodies they have the mark of the witch. …Burn the two witches at the stake because by doing so you shall be able to burn all ye own sins against horses*

[25] (p. 270).

The parable of Cambridge and the other two stallions as helpless victims of mercenary, foreign witches who could be saved by media commentators unfamiliar with him was bolstered by decontextualised, illustrative newspaper use of large black-and-white and colour images of Cambridge. In a media report titled “Dark Horse Outrage”, it was claimed that ‘for at least two years the three highly strung stallions have lived on hard concrete in their cold, brick stable’ [29]. Accompanying the two-thirds page article were four images, one of Cambridge lying on his side, asleep in his box. For an unknowing audience, ‘the image of the animal and accompanying headline can be considered as contributing to a discursive struggle in defining who is humane and who is inhumane’ [30] (p. 64). In the *Werribee Banner*, pro bono lawyer Carolyn Burnside reiterated advice provided to her concerning Cambridge’ state of health: ‘If the horse is groaning, clearly it’s in pain and suffering’ [31]. In another report, the protest group organiser claimed that ‘Cambridge was lying down and breathing as if he was suffering asthma’ [26].

A week later, these representations were given an appearance of legitimacy when RSPCA national president Dr. Hugh Wirth claimed that Cambridge was ‘confined in darkness’, ‘in constant pain’, and that ‘the most humane thing now is to destroy him’ [32]. In the same report, titled “Vet Says He’d Shoot Sick Horse” ‘a furious Dr. Wirth disputed claims that veterinary experts were in charge of the horse Cambridge’ and began ostracising the consulting specialist veterinarian, whom he described as ‘not a registered horse specialist but is an academic with a special interest in horses’, before stating: ‘One has to question [the owners’] motives in preventing two inspectors and three vets expert in equine care from inspecting their animals’ [32]. Unacknowledged by the RSPCA spokesperson, was that such an inspection was not authorised by Section 6 (1e) of the Victorian Prevention of Cruelty to Animals Act (POCTAA) which exempts ‘the treatment of any animal for the purpose of promoting its health or welfare by or in accordance with the instructions of a veterinary practitioner’ [33] (p. 11). The legislation prevented interference by RSPCA Inspectors because they lacked the expertise to contribute to Cambridge’s care in any meaningful way. Subsequent threatening representations on Melbourne radio necessitated further legal action through a Victorian Supreme Court Injunction to restrain Executive Officer Kevin Apostiledes or any RSPCA inspector from ‘killing, touching, or otherwise interfering’ with Cambridge [34]. Following this, the RSPCA requested the Veterinary Practitioners Registration Board of Victoria (VPRBV) to investigate the ‘appropriateness of the treatment’ of Cambridge being administered by Dr. Horsey, on the ground that ‘“Cambridge” is being used as a research vehicle for Professor Chris Pollitt in his search for a cure for this issue of Laminitis’ [35].

While Dr. Wirth refused to accept Dr. Pollitt’s veterinary credentials, contributors to Cyberhorse stereotyped him as a Frankenstein-like unethical veterinary researcher pursuing weird science at the expense of the horse [27]. Eventually ‘the plight of the horses reached Parliament … when Opposition agriculture minister Steve McArthur claimed Victoria’s prisoners were treated more humanely than the stallions’ [36]. The human and animal comparison that is often used in animal advocacy draws here on a speciesist logic that humans have more worth than animals, with the implication being that if the animal-like prisoners in Victoria’s prison system are being treated more humanely than the valuable horses in question, then there is something seriously amiss in the natural order of things. Francesca and Allison Raffaele were also conceivably animalised with commentators describing them as ‘Sly as foxes’ [25] (p. 267) and ‘prey animals’ [25] (p. 274), with calls to ‘put them down’ [25] (p. 339). The positioning of criminality in media representations consistently drew, ironically, on a speciesist figurative logic of humanness vis-à-vis animality. This logic is based on a widespread understanding of animality as a constitutive dimension of how criminality is discursively constructed. As Jason Wyckoff explains: ‘Animality involves intersecting features of animals’ lives, features which are constructed according to the ways in which particular animals or classes of animals are situated within human institutions and practices’ [37] (p. 545). Our linguistic practices and our legal institutions both play a role in the discursive construction of both animals and certain classes of humans as subordinate, which reinforces the linguistic practices and legal institutions themselves [37] (p. 545).

Another media article titled “Expert Distressed by Stallions’ Stalls”, cited ‘Australia’s leading expert on animal behaviour, Andrew McLean’, who described the stallions’ behavioural response to their housing in the barn at Werribee Park as ‘tragic’, claiming that the horses have kicked out forcefully and repeatedly, which is a classic sign of chronic stress’ [38]; “chronic” inferring that the stallions had been imprisoned for a prolonged period. Copenhagen and Congressman were removed from WPEC within 24 hours of Cambridge’s euthanasia, after I as a precaution, organized for full veterinary clinical examinations which revealed ‘both were normal, sound, and in good body condition’ [25] (p. 347), contravening claims that the stallions had suffered in their confinement. McLean’s assertion that the stallions had repeatedly kicked walls to signal their powerlessness, which cannot happen without sustaining injury was, however, speculative.

Kay Weaver remarks that ‘the highly emotive nature of discourses around the treatment of animals … contributes to their newsworthiness. Stories are highly emotive when they involve the shocking, the unexpected, [and] the immediate’ [30] (p. 62). Public ostracism, including cyberostracism is an interpersonally aversive behaviour which aims to marginalise its target, thereby threatening an individual’s sense of belonging, self-esteem and feelings of control over their environment. Discourses which construct individuals as outsiders can lead to ‘them being thought of as not having humanity’ [39] (p. 161). Once an individual is dehumanised, imputing a capacity for animal cruelty is then within the realms of believability. In this sense, as Stuart Hall observes ‘the media are not simply institutions that reflect consensus’ but also ‘produce consensus and “manufacture consent”’ [40] (p. 432). ‘Importantly’, states Dina Gavrilos, ‘the news media accomplish this because of their own ideology of impartiality and objectivity, which gives the news its cultural basis of credibility’ [40] (p. 432).

The autoethnographic vignettes that follow posit a counter narrative that explores Cambridge’s training and treatment through self-reflexive, phenomenologically aware praxis. As such, the developing interspecies relation between owner/trainer is described, interpreted, and analysed from a personal point of view. The vignettes are necessarily selective, and are focussed on Cambridge’s birth, his early years and dressage training, and the co-created agentivity that shaped he and his owner’s responses to veterinary treatment for laminitis, which was at the time a relatively opaque disease, about which there were conflicting accounts of causes and treatments in the scholarly literature [41]. 

## 3. Meeting Cambridge

On a chilly October night in 1990, I huddled in a corner of the foaling box with my coat collar pulled up and beanie tugged down, to await the arrival of Selket’s much-anticipated foal. She’d been through the early agitations and was now prostrate, grunting and blowing, as she succumbed to an irresistible urge to push. A head appeared, two white legs now projecting like knitting needles anchored in a ball of wool. The leg joints were big, which had me expecting a protracted, assisted foaling, but her efforts built in a quick crescendo, and she expelled the foal herself in a rush. Selket remained prostrate, her flank spasming, ragged breaths shooting from her nostrils, ears now forward, now back. I moved in close to draw the birth sac back from the foal’s head, clearing the nostrils. Sitting up immediately, the colt was breathtakingly chiseled. I longed to touch his glistening claret coat, but an air of superiority about him sat me back on my heels. Shaking fluid from his ears and chin, the colt trembled with life, then began stomping outstretched forelegs on the straw. His shrill whinny pierced the quiet, Selket stirring to gaze at her baby, nostrils quivering, a nicker barely audible. I was pleased that she remained reclined so that oxygenated blood could continue to pulse through the umbilicus, but the colt had other ideas. Yanking his hindlegs free, he drew them underneath him. And then, within minutes, in a sweeping singular act, he raised himself up off the straw bed, and stood swaying slightly, his balance never in doubt. It was a rare and extraordinary demonstration of athleticism and strength. Watching in awe, I sensed a shift in the natural order of horse in human world: what in time became an overwhelming presence, disarmed my inclination to imprint him with my humanness. Cambridge had, in his first minutes of life, begun defining the terms of our relationship.

**Figure 1:**
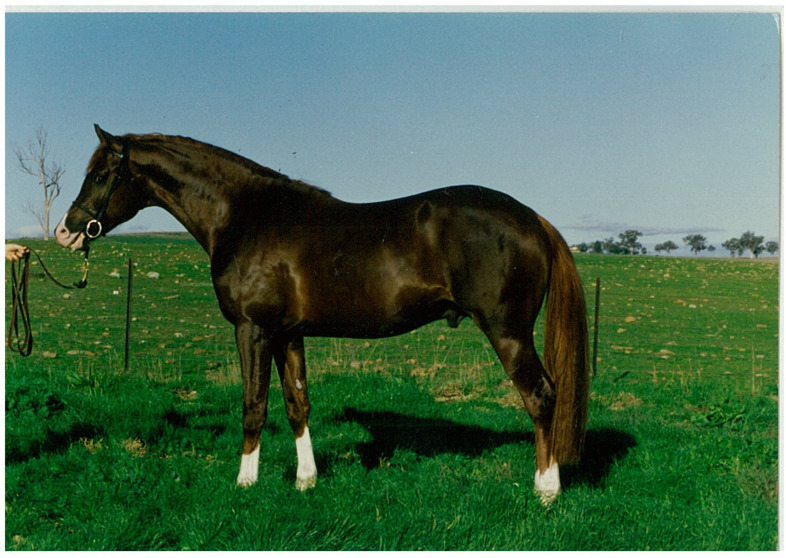
Cambridge at 2 years.

Over the next two years, Cambridge was weaned and sparingly handled, limiting the humanness imprinted on him. He learned quickly, and significantly, he resisted becoming the object of my affections. One night he fled his box in a dramatic thunderstorm, in its aftermath regarding me, *his* human. But that didn’t make him affectionate towards me; Cambridge was a serious, even distant horse, and I learned to be content with admiring him from afar. Others, including internationally prominent industry figures sensed Cambridge’s reticence too. A big horse, Cambridge was regarded as without fault. He was perfect, physically, at least (Figure 1).

## 4. Learning Cambridge

As a 3yo in mid-1994, Cambridge was ready for “breaking-in”, a term which refers to those actions taken by a trainer to subdue the horse to be ridden by a human. Breaking-in is a forever, ongoing experience as suggested by the continuous present tense of the word ‘breaking’. It should not mean *broken.* I didn’t anticipate with Cambridge, however, that altering our spatial proximities would be met with resistance: Cambridge refused to be ridden.

Parsing the intricate language Cambridge and I developed over years of working together following this initial incident, is beyond the scope of this paper. But to summarise, when I realised that Cambridge would not let me sit astride on him, I entrusted him to an expert showjumper and thoroughbred trainer/breaker-in. After three short weeks he returned to me, violently resistant to being ridden. This focus on Cambridge as transgressor, however, denies interspecies entanglement or in furtherance to de-centering the human, as ‘more-than-human’ encountering, which ‘highlights the intertwining of heterogeneous entities. As entities converge in this way, any notion of authority becomes ambiguous… humans cannot be assumed to be in control’ [42]. With the breaker, Cambridge’s fright-flight response was countered using a hobble strap, which forced him to stand on three legs for the mount [25] (p. 31).

After an extended period of time, during which I hoped Cambridge would forget the psychological harm incurred by the breaker, I sent him to the training facility of an expert behaviourist/educator, believing that he could establish a rapport with him, but instead, he found Cambridge “intimidating”. Cambridge resisted his training and management regimens, and was again, worse for the interaction. Following on from the failure of behaviourism’s learning theory as a model for Cambridge’s re-education, and from the unavailability of an alternative training facility which would allow me to manage his daily needs, I relocated him in 1996, to WPEC—a place where Cambridge and I could figure things out together and I could care for him. We’d lost many months and there was now no time to spare. As a venue for competition and industry events, the centre was still underused at that time. Agistment was not offered per se, the arrangement for Cambridge an exclusive one. There was an enormous 80 × 30 m indoor arena and a single box, in isolation, available, recessed within a high-gated shed. It was light and airy, with a tree at the back to keep it cool. Unassuming. Somewhere to start over. Just Cambridge and me. Aside from a two-week period recuperating from an eye injury, in which veterinary instruction required Cambridge to be sheltered from natural light in a deliberately darkened box, he was safe, content, and very much focused on his work. It was during this short interval though, that an equestrian patron mistakenly assumed that a stallion—Cambridge—lived in darkness, the assumption being central to the media misrepresentations of cruelty.

However, with the regimens of two predominant schools of equine education, traditional and behavioural, resisted by Cambridge, I was forced to disregard all established industry knowledge and turn instead to the horse himself: allow Cambridge to teach *me*. As philosopher David Wood observes, that ‘once we have seen through our self-serving anthropocentric thinking about other animals, we are and should be left wholly disarmed, ill-equipped to calculate our proper response’ [43] (p. 32). I forwent any attempt to ride Cambridge and worked with him to build a rapport in groundwork instead, walking alongside him and applying precisely applied pressure at his girth instead of a rider’s heel. Timing was crucial. If I lost concentration and mistimed the cue, compromising Cambridge’s natural swing, he responded with feral agentivity, swiftly but gently pushing me into the wall skirting the arena, and holding me there for minutes to ensure—I contend—that I understood my actions had consequences. Cambridge was teaching me what Donna Haraway [44] (p. 244) has coined ‘becoming with’: ‘[i]f we appreciate the foolishness of human exceptionalism then we know that becoming is always becoming with, in a contact zone where the outcome, where who is in the world, is at stake’. Cambridge would not tolerate power *over*, teaching me instead, what is meant by power *with*. When many months later, I sought to ride Cambridge, he offered no resistance to being schooled in dressage, provided I respected both his natural swing (abstaining from concurrent leg pressures) and his contribution to the training process (determining the progression of gaits and higher movements), which acted to abolish industry-acceptable ‘implicit consent, in that horses learn to cooperate (or not to challenge) with humans’ [45] (p. 135). I believe that in these key tenets lay the answers at all stages of the breaking-in and education process—which are inherently violent—to equine acquiescence without human duress. This nascent relation of power was to later flourish as Cambridge transformed from athlete to patient.

## 5. Nursing Cambridge

In an entirely unaccountable event, on 4 May 2000, Cambridge experienced an acute episode of laminitis. I found him in his box standing with head lowered, fore feet placed out in front of his body, with his weight drawn back over his hindquarters, and sweating profusely. I phoned veterinarian Dr. Horsey, who diagnosed laminitis, a disease I was unfamiliar with. After questioning me about Cambridge’s diet and exercise—possible domains of causation—and being satisfied that there had been no fault with either, Dr. Horsey administered a potent mix of analgesics, anti-endotoxics, and anti-inflammatories and was able to do so despite Cambridge being ordinarily violently averse to being needled.

That evening, Dr. Horsey returned for radiographs, Cambridge having to stand on wooden blocks for an unimpeded view of each hoof. Cambridge could not bear his weight on one foot, to place the other on a block, and he was fearful of the hollow sound made when his hoof contacted the timber. Two long hours later, we had x-ray plates. Dr. Horsey did not expect Cambridge to survive the night. I curled up in a corner of Cambridge’s box to keep vigil, and in the early morning hours he lay down and slept.

The next morning, Cambridge was much improved, but radiographs confirmed rotation of the distal phalanx of both forefeet. After a brief explanation of the disease’s pathology, Dr. Horsey’s prognosis precluded any return to full soundness. Knowing that I had competition aspirations for Cambridge, he said, “this is an absolute catastrophe” [25] (p. 89). He explained that in most instances, horses are euthanised after contracting laminitis, either immediately, or when an owner chooses not to pursue the recovery process, due to it requiring a lengthy time frame, a large financial investment, and a commitment to daily care under the guidance of a veterinarian and farrier [46]. I didn’t hesitate to pursue recovery, feeling that with meticulous application, Cambridge could be saved. Dr. Horsey and I discussed how to tackle Cambridge’s treatment, with pharmaceutical and alternate treatment options, remembering always to respect Cambridge’s agency in the recovery process: Cambridge was very ill, but his mind was sharp, and his manner, discriminatory. To accommodate Cambridge’s resistance to being needled, we opted for powders, pastes, or pellets instead. I replaced the straw in Cambridge’s box with a 30 cm packed bed of wood shavings as recumbency was the goal: relieving the weight-bearing of his front feet. He was necessarily administered pain relief daily, in the form of phenylbutazone. However, long-term administration of this NSAID anti-inflammatory is known to damage the stomach lining in horses, a factor compromising protracted recovery. In my various readings, I noted that ascorbic acid may be beneficial in this regard, so it was added to Cambridge’s daily regimen. And with Dr. Horsey’s agreement, I sought out a specialist, Dr. Chris Pollitt, one of two veterinarians considered to be world experts on the disease. Fortunately, Dr. Pollitt was the Associate Professor in Equine Medicine at the University of Queensland, steering the Laminitis Research Institute, with an extensive publishing record as a researcher into the pathology of equine laminitis and its treatment.

During Dr. Pollitt’s initial consultation, with the assistance of his text, *The Colour Atlas of the Equine Foot* [47], he explained in minute detail the disease’s pathophysiology. For our purposes, laminitis is described as:

*the failure of the attachment between the distal phalanx (coffin bone) and the inner hoof wall. A horse has laminitis when the lamellae of the inner hoof wall, which normally suspend the distal phalanx from the inner surface of the hoof capsule, fail. Without the distal phalanx properly attached to the inside of the hoof, the weight of the horse and the forces of locomotion drive the bone down into the hoof capsule, shearing and damaging arteries and veins, crushing the corium of the sole and coronet, and causing unrelenting pain and lameness’*

[48] (p. 188).

Dr. Pollitt also enquired about Cambridge’s diet and exercise and we discussed changes in Cambridge’s environment. I summarised what had transpired over several months when the barn was filled with polo ponies. One mare repeatedly escaped her box to seek the attention of the all-stallion bachelor group, causing Cambridge, an experienced breeding stallion, considerable distress. Cambridge’s gait irregularities first became noticeable during this time, and according to Dr. Pollitt, a bruised sole, which had halted Cambridge’s training, signaled the start of the disease. Had I realised this, I would have hastily relocated Cambridge to a property where my other horses were, as the needs for recovery were quite different than those for training. By the acute episode in May, however, Cambridge’s safe removal was no longer possible.

Dr. Pollitt became our consulting specialist and either performed critical trimming of Cambridge’s hooves himself or worked alongside master farrier, Richard Caldararo. He travelled from interstate to assess Cambridge on average, every six to eight weeks throughout the recovery, always making himself available by telephone, even when overseas. Dr. Horsey, or an associate, attended Cambridge on average twice weekly. His response to the RSPCA complaint lodged with the VPRBV, contained hundreds of hand-written and computer-generated invoices, drug dispensing records, veterinary inspection reports, blood analyses, faecal occults, ultrasound scans, and 25 radiographs obtained throughout the treatment period [25] (p. 390). However, media reportage insinuated that veterinary treatment was inadequate, hence the negativity surrounding the invocation of Clause 6 (1)e of POCTAA [33] (p. 11) to protect Cambridge from unlawful threats of the RSPCA, the welfare body casting Cambridge as a victim of his owners’ financial interests and Dr. Pollitt’s laminitis research.

From the outset, WPEC did not support Cambridge’s recovery, insisting that he be located elsewhere. Dr. Horsey advised that transportation would likely incite further rotation and displacement of the distal phalanx of each fore hoof, eventuating in Cambridge’s euthanasia. When WPEC engaged Dr. Patricia Ellis for a second opinion, she unequivocally supported Dr. Horsey’s assessment. However, WPEC did not accept her advice, quite possibly because a prominent WPEC board member believed that Cambridge wasn’t even ill, insisting that “if he [Cambridge] had laminitis, he’d be on his back with all four legs in the air” [49]. For the unknowledgeable observer, Cambridge, due to the treatment protocols, exhibited no external signs: he was a picture of health. Upon instruction, I escalated to legal remedy, an Interim Injunction in the Victorian Supreme Court, to prevent WPEC forcibly removing Cambridge. Court Orders by consent provided for Cambridge to remain until such time as Dr. Horsey or Dr. Pollitt deemed him safe to travel, and for two of his sons, Copenhagen and Congressman, to remain as companions, one stallion remaining boxed alongside Cambridge whilst the other was exercised [50]. Unfortunately, despite WPEC management agreeing with the orders, from that moment previously received advance notice of environmental disturbances was withdrawn, leaving Cambridge vulnerable. Dr. Horsey introduced a low daily dose of acetyl promazine, a mild sedative which was later adjusted during industry events to encourage recumbency, the relief of weight bearing on laminitic feet being a key objective for recovery. Dr. Horsey was guided by Dr. Pollitt’s prognosis of Cambridge’s laminitis; within a few visits, due to responsiveness to treatment, Dr. Pollitt advised that although a competitive career was highly unlikely, training soundness was possibly achievable [25] (p. 117). This advice encouraged me to proceed, as the knowledge that I’d gained with Cambridge in training was worth pursuing in itself, in light of its difference to existing training paradigms. The recovery did become protracted, progressing remarkably in response to treatment protocols and regressing in response to the many environmental aggravations in his immediate vicinity. Cambridge’s box, his *safe* place, became compromised.

Throughout the treatment period, the veterinarians evaluated Cambridge’s health in accordance with the principle of ‘quality of life’, an assessment protocol supported by McGreevy et al. [51] (p. 3), in their readings on the subject [52,53,54,55] who advise that expert opinion is the most suitable and reliable determinant. While there was an expectation for breeding Cambridge, he was never solely a financial investment as the media claimed. Cambridge was also valued intrinsically, meaning ‘that something has value in and for itself and that, in principle, it should not be treated only as a means to another’s end’ [56] (p. 143). Hall et al., claim that ‘horse-human interactions undoubtedly influence both the subjective emotional experience and the behavioural expression of the horse’ [57] (p. 184), reflected in veterinary reports attesting to Cambridge’s health and the corporeal reciprocity experienced between him and the treatment team. But aside from these anthropocentric paradigms of valuing and possibly euthanising Cambridge while recovery was feasible, euthanasia would have meant denying an extraordinary stallion his agency, manifested in adaptations to circumstances, such as cooperating with the meticulous farriery which necessitated him standing absolutely still for long periods (without the veterinarians resorting to heavy sedation or nerve blocks or any form of additional restraint), while frog support moulds hardened, or layers of Kevlar wrap basted in the adhesive, Equilox, matured.

About six months into treatment, radiographs were required of the left fore. Cambridge had to stand on the wooden blocks again. Noticeably uncomfortable standing on the concrete, Cambridge shifted his weight from one foot to the other as Dr. Horsey positioned the blocks. When I gave the lead a gentle tug, Cambridge resisted, tugging back and releasing my grip, so as to haul his weight back towards his powerful hindquarters in a levade, the front half of his body levitating as he gently and precisely placed both forefeet on the respective blocks. This was a remarkable response to which after a moment of disbelief, everyone exhaled.

Cambridge’s action was a demonstration of adaptive innovation, a ‘process that generates in an individual a novel learned behaviour that is not simply a consequence of social learning or environmental induction’ [58] (p. 395). He had only the once prior, stepped onto the blocks for radiographs, which suggests that it was Cambridge’s memory of the associated pain that prompted him to approach the task differently. Viewing Cambridge’s behaviour through a phenomenological lens, which in the first instance, privileges the experiential over the theoretical, and given that his response was immediate and expertly performed, it is quite possible that Cambridge had exhibited prospective memory, which is ‘remembering to execute intended actions at the appropriate moment in the future [and] is an important cognitive ability’ [59] (p. 40). However, prospective memory is yet to be established in equines within a scientific framework. For me, the moment was pivotal because it reminded me that I could only ever make suggestions to Cambridge. Ultimately, he’d have the final say. 

After an incident when Cambridge was distressed by a tractor slashing grass immediately outside his box, wrenching a shoe fastened with Equilox from his hoof—which led to displacement of the distal phalanx and pronounced abscessation—I began heavily strapping both forelegs and feet for protection and support, despite having to remove and dispense with the dressings daily. I did this in the evening when we were least likely to be disturbed, as Cambridge was not sedated, and the two-hour process could not be stopped once commenced. I began by carefully driving a scalpel blade through the outer layers of self-adhesive bandaging, tugging downwards to the bottom of the hoof and then gently prising open and removing what effectively was a cast. Cambridge stood unmoving as I unwrapped the inner layers of cotton wool to expose his foreleg, the sculptured beauty of it almost always making me pause. I gave his leg a hearty rub before lifting it to slip off the pungent dressings, now damp with drained fluid. Then I bathed the hoof with warm, salty water, Cambridge dropping his head to nuzzle my hair.

The ordinarily seamless junction from horn to skin (coronary band) had ruptured across the toe, its edges swollen and ragged. The split would form a scar ridge in the wall as it travelled downwards over time. I placed paraffin gauze swabs over the area, then picked up Cambridge’s foot to encase it in layers of cotton wool and a liner. It was a precarious moment, with Cambridge’s foot resting upside down on my bent knee, dressings strategically placed, and me whizzing a light, self-adhesive bandage around it before he placed his foot down for relief. Sometimes, I wasn’t quick enough, dressings scattered, and sawdust flicked over his exposed hoof.

One evening following the commencement of abscessation as I prepared dressings, Cambridge lay on his sternum, in ‘mind’ sleep. Deep in dreams, a muscle twitched, or his head wobbled, or his exhaling breaths became grunts or groans. Media reports, including the above-mentioned *Werribee Banner* report in which lawyer Carolyn Burnside equated Cambridge’s sleep behaviour as indicative of pain [31], were mistaken. In my experience, horses do not respond to pain as humans and some other animals do. Regardless of whether an injury, illness, or disease is life-threatening, horses generally suffer pain in silence, with profuse sweating and an elevated heart rate, reliable indicators of distress.

Eventually, Cambridge woke up but did not attempt to stand. As it was very late, I gambled on a dressing change with Cambridge recumbent, which if he acquiesced, would eradicate the discomfort of standing on a compromised hoof. I knelt next to him, slipping on his bit and his training bonnet—I worked him in a half-cup racing bonnet—and then sat with him to gauge his response. Cambridge lay there, unperturbed. Allison replaced me at his head and, incredibly, Cambridge rolled onto his side. I interpreted this—as I had done during Cambridge’s training days when he did something unexpected—as him encouraging me to follow his lead. In a moment of intersubjectivity, horse and humans were reflecting what Elizabeth Behnke terms ‘grounding’, which ‘alters the ‘motor intentionality’ of the situation, for letting weight settle interrupts the bodily “being on the brink of” reacting in a particular way’ [60] (p. 103). When I finished, Cambridge sat up. I quickly cleared the box making room for him and he rose to his feet. From that night forward, I changed Cambridge’s dressings with him recumbent. The unconventionality of this arrangement cannot be overstated: Cambridge was not a powerless victim; he was an empowered participant in his recovery.

**Figure 2:**
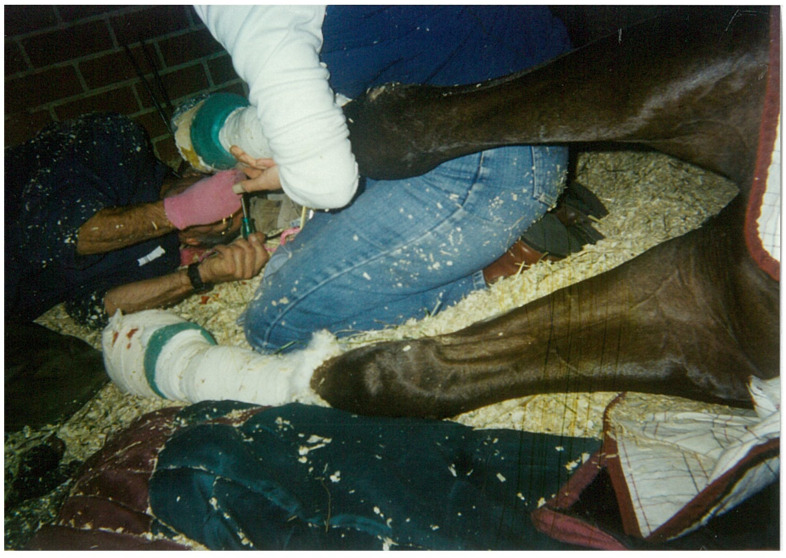
Pollitt, Christopher. *Co-created agency: Cambridge, Francesca, and Richard Caldararo refitting a shoe*. 2001. Digital image. Personal communication.

When Dr. Pollitt next returned to monitor progress, I decided that we should attempt a trim with Cambridge recumbent, knowing that it could mark a significant loss or gain for his treatment. Cambridge would extend his trust to the full team or possibly withdraw it altogether. And even if he did acquiesce, it was a big ask of the veterinarians and farrier, and not without considerable risk. But as I peeled away the cast and foot dressings, the farrier squatted next to me, then lay in the bedding to inch his way under Cambridge’s foot, tapping it lightly with his hoof knife. Cambridge did not react. With me suspending the leg, the farrier chipped away the Equilox adhesive to remove the shoe (Figure 2).

Dr. Pollitt angled in beside him, both with the wall pressing at their backs. Cambridge could be heard grunting, the sound signaling relaxation. As Suvi Satama and Astrid Huopalainen note, ‘caring for an animal is about constantly negotiating non-verbal, bodily interactions in which animal agency comes into being’ [61] (p. 366). In time, Cambridge would fall asleep and snore, when the treatment team was present in his box. A lump formed in my throat as I felt momentarily transported from the scene, watching from above. The veterinarians and farrier, all senior in years, eminently qualified and respected, and denied their usual and expected conveniences, were forging an unprecedented relationship with a horse who continued to meet them at the interface of interspecies engagement, despite the harms he had suffered at human hands. In a paper titled, ‘The 2020 Five Domains Model: Including Human-Animal Interactions in Assessments of Animal Welfare’, agency is described as ‘apparent when animals engage in voluntary, self-generated and/or goal-oriented behaviours’ [62] (p. 13). While Cambridge appeared to embody this description of agency, the scene suggested something more than a demonstration of autonomy. It was collaborative, or what Gilles Deleuze termed *agencement*: ‘a rapport of forces that makes some beings capable of making other beings capable’ [24] (p. 38). Cambridge and his team were affecting and being affected within an atmosphere of co-created possibilities, in which ‘the *agencement* resists being dismembered, resists clear-cut distribution’ [24] (p. 38).

Recumbent for two hours, Cambridge rose immediately to his feet on completion and was eager to eat. When he lay down again, he swapped sides, providing ready access to the left foot, a convenience not wasted on the treatment team. When the long process was complete, Cambridge stood up, shook himself and returned to his hay net, eyes bright and ears pricked. The farrier leaned against the grill beside me to watch him. “If ever a horse deserved to live” he said, “it’s Cambridge.” [25] (p. 172). The farrier explained that he had been to laminitis clinics in the States. Horses do have their feet trimmed lying down, but that’s because they *can’t* get up. In a later conversation, Dr. Pollitt commented that he had never experienced or heard of a horse responding the way Cambridge had; Cambridge was truly remarkable.

From that treatment onward, Cambridge always swapped sides regardless of which side started on, curtailing conceptions of coincidence. Behnke posits that if we can accept that anything we do with an animal without their permission is a violent act to some extent, then a key tenet for interspecies trust and cooperation rests with interactions in which there ‘is no longer a question of either “actual” or “averted” violence, no longer an occasion for “potential” violence, but an occasion for the practice of peace’ [60] (p. 110). In Cambridge asserting his subjectivity, rather than being the object of ours, we became co-participants or companion agents in:

*a fluid situation—not only one in which “what will happen next” is not determined in advance, but one in which “what kind of situation this is” is open in principle to transformation’; it is an openness which ‘allows “something else” to emerge*

[60] (p. 110).

As the recovery progressed, Cambridge continued to defy stereotypical representations of a horse disabled by chronic laminitis (crippling hoof pain, permanent recumbency, and depressed mood) and associative phenylbutazone toxicity (oral and gastrointestinal ulceration with subsequent muscle wastage) which underpinned claims of his suffering in the media. Cambridge’s adaptations to his ongoing treatment exhibited what appeared to be overtly agential behaviours that seemed uniquely designed to assist his humans with their care regimes. For example, ordinarily, horses rise to their feet frontend first, sitting momentarily on their haunches with forelegs extended in front of them, then pushing back against the body with the hindlegs drawn in underneath, before rising behind. This procedure allows them to quickly scan their surroundings for perceived threats before then springing into action if necessary; an instinctual behaviour defined by ethologists as ‘revealed through uniform, standard performance movements’ [63] or ‘behaviour patterns [which] are rigid and cannot be modified by experience’ [64] (p. 579). Innate or instinctual behaviours are referred to phenomenologically as “biological drives.” McGreevy claims that ‘most of the published peer-reviewed data strongly indicate that horses lack insight into their instinctive behaviours’ [65] (p. 287). This implies that a competency for innovation, more particularly, invention, would be necessary to contradict these claims. Grant Ramsay, Meredith Bastien, and Carel van Shaik explain that ‘cognitively complex innovations reflect the presence of causal reasoning, correspond[ing] more to inventions’ which ‘never arise by accident, simply because the motor acts involved are highly unusual, and deviate rather strongly from the rest of the motor repertoire’ [58] (p. 406).

When Cambridge altered to rising to his feet backend first—rising rump high onto bent knees, then hauling his bulk back over his hindquarters to minimise weight from the shoulders forward, levitating with his hocks close to ground level, forelegs stretched into air in order to then place his front feet gently on the bedding—he not only defied expectations of disablement but also demonstrated what appeared to be insight into his biological drives. He did not achieve this clumsily through ‘ABC learning: that is, [animals] come to modify (or re-design) their behaviour in appropriate directions as a result of a long, steady process of training or shaping by the environment’ [66] (p. 87), but as an abrupt amendment. Following this, Cambridge reverted to an instinctual response only when frightened to his feet, having been denied preparatory thinking. This modification to species-specific behaviour is apodictic, requiring us to reconsider Konrad Lorenz’s ‘strong tenet that instinct and experience are exclusive’ [64] (p. 582) and that ‘behaviour is to be explained by means of its underlying physiological causal basis, but not by means of invoking psychological drives and subjective motivations of behaviour’ [64] (p. 584). Cambridge appeared to be motivated in this moment and previously in relation to the x-ray blocks by the avoidance of discomfort in his front feet. He did not fall into permanent recumbency but with dignity he insightfully altered a behavioural pattern to accommodate his need. But something must also be said for agencement here, because Cambridge did not accept a state of powerlessness consequent upon his vulnerability in relation to human-centred dominance but was a participant in a co-created relationship characterised by openness, trust, and respect with the humans dedicated to his recovery. He had not learned helplessness but displayed agentivity in acting with initiative.

There is no question that from any objective standpoint, harms had been inflicted upon Cambridge, both in his breaking-in and re-education with traditional and behaviourist methods, respectively, and in the conditions pertaining to his housing (loose mares in oestrus, environmental disturbances from machinery inside and immediately outside the barn, and a general lack of consideration from staff and industry patrons particularly during busy and/or prolonged events) in what was otherwise considered an elite venue. However, the truth is rarely simple and the media misrepresentations of Cambridge as helpless victim did not consider whether or not Cambridge may have wanted to heal, and that like most sentient animals had not only an interest in not suffering, but also had an interest in remaining alive, and that he adapted instinctual behaviours to assist in his recuperation.

When Dr. Pollitt attended Cambridge on 5 June 2002, to inspect and trim his feet, he did not envisage euthanasia, but the first trace of osteomyelitis, an often complicating and untreatable factor in chronic laminitis which we had prevented for 24 months, was evident in the left fore’s distal phalanx. For this reason, only, and with great sadness, Cambridge was euthanised. A veterinary clinical examination conducted prior to his death reflected his general health and wellbeing with no noteworthy abnormalities present, and a blood test indicated all markers within the normal range. Dr. Horsey was confident that the ascorbic acid given to Cambridge each day, had resisted the side effects of long-term phenylbutazone administration. A later study focused on gastritis and gastric disease in humans found that ‘ascorbic acid plays a key role in healing and protection of the gastric mucosa from injurious insults’ [67] (p. 2512).

## 6. Defending Cambridge

In his response to the RSPCA’s complaint of cruelty towards Cambridge, Dr. Horsey was confronted by the need to justify his ongoing treatment, being responsible for monitoring Cambridge’s health. Very late in the treatment period, he stopped by one evening when his busy day had ended, to deliver dressings. We stood at Cambridge’s door, watching him pull hay from the net at the rear of his box. Cambridge turned to look at us, then made his way over to affectionately nuzzle Dr. Horsey. It was the only time that he’d reacted to Dr. Horsey, and in such a deliberate manner: not the powerful/powerless binary depicted by the media, but a moment of interspecies intimacy. I know that it meant a good deal to Dr. Horsey, because despite hovering on the perimeter when Dr. Pollitt visited, he’d availed himself at all hours for his number one patient. We didn’t know it then, but it was the last time Dr. Horsey would see Cambridge as Dr. Horsey was shortly afterwards hospitalised for hip replacement surgery. Of course, an animal cannot know these things. Or can they? Cambridge sensed something that caused him to act in a way most unlike him; something that prompted him to wordlessly gesture, what I interpreted as gratitude.

Throughout the intense period of media reportage, the RSPCA’s spokespersons had expressed dissatisfaction with the society’s inability to overrule the veterinarians’ legislative authority as they sought to enforce an inspection of Cambridge by the welfare body’s team of veterinarians (of which none of them were experts in laminitis) and animal inspectors. Any inspection of Cambridge’s feet would not have occurred without Dr. Pollitt and Dr. Horsey present. The inclusion of the RSPCA’s team implied that nine humans were to be present in a very restricted space, five of them unfamiliar with the treatment protocol, and each of them strangers to Cambridge, with a ‘body which moves, which walks, bears and diffuses smells, makes noise, follows, and everything a body may do—including what we don’t know our body may do since we are so unaware about what it is capable of, but which animals may, nevertheless perceive’ [68] (p. 52). To have allowed an additional five strange humans to enter *his* box, assessing him from a singular, visual perspective, standing up or at most, stooping, as a collective ‘theorising spectator’ [60] (p. 95) would have most likely been perceived by Cambridge as a threat. He knew and trusted only the humans of his treatment team who, when in his box, transcended to a collective bodily receptivity and proprioceptive awareness. An inspection would have necessitated anaesthesia: in these circumstances, a violent act bypassing Cambridge’s consent. Cambridge was not *something* we did things to; he was *someone* we did things with.

Certainly though, with the “rule of thumb” in the Australian horse industry for the treatment of laminitis being understood as ‘putting the horse out [to] pasture, see if he comes good, and if he doesn’t you have to put him down’ and that long-term administration of phenylbutazone will ‘just the eat the horse’s guts out, so you’re going to end up with a dead horse anyway’ [26], the stereotyping of Cambridge as a candidate for euthanasia prohibited an educative opportunity for veterinarians, the equestrian industry, and the general public concerning the disease and its necessary protocols for recovery. Weaver [30] (p. 63) explains that ‘while news reporting of animals draws on a range of discursive frameworks and narrative traditions, the question remains as to why particular discourses around animals are privileged over others, and who benefits from those privileged discourses? Examining this issue requires identification of the sources for news stories about animals, as well as the agendas of those sources’.

## 7. Remembering Cambridge

Stakeholders who contribute publicly to animal welfare debates can hold conflicting values and viewpoints on what animal welfare is and how a good life for a particular animal might be achieved. These views will be conditioned by conscious and unconscious factors, level of education and demographics, and agendas that inform their ethical reasoning in relation to moral choices in particular animal welfare cases. Such interventions can signal variant approaches to welfare problems, or problematise specific aspects of animal welfare, and propose different corrective actions, whether by rational assessment of ‘quality of life’ according to the Five Domains model, rights-based determinations based on sentience or subjective value-laden views about ‘significant otherness’. Philosophical and ethical considerations aside, the narrative and argumentative discourses that framed media representations of Cambridge’s confinement and its implications, including calls for his euthanasia, were not only based on dubious sources and hearsay, but also a failure to consider whether or not the horse had a point of view. While the RSPCA’s calls for averting Cambridge’s suffering by euthanising him were anchored in particular interpretations of soft regulations and guidelines on euthanasia in Victoria, assertions about ending a horse’s life without ever having met the animal suggests an anthropocentric confidence in the right to pass judgement on an animal’s quality of life and mandate his/her death as a right in itself, ignoring in this instance Cambridge’s instinct to not die. When the complexity and singularity of an animal’s desire to live is denied, violence against them becomes normalised, and institutionalised, as in legal codes that protect or exempt from violence certain classes of animals, not by their capacity to suffer, but by their value or use to humans. 

**Figure 3:**
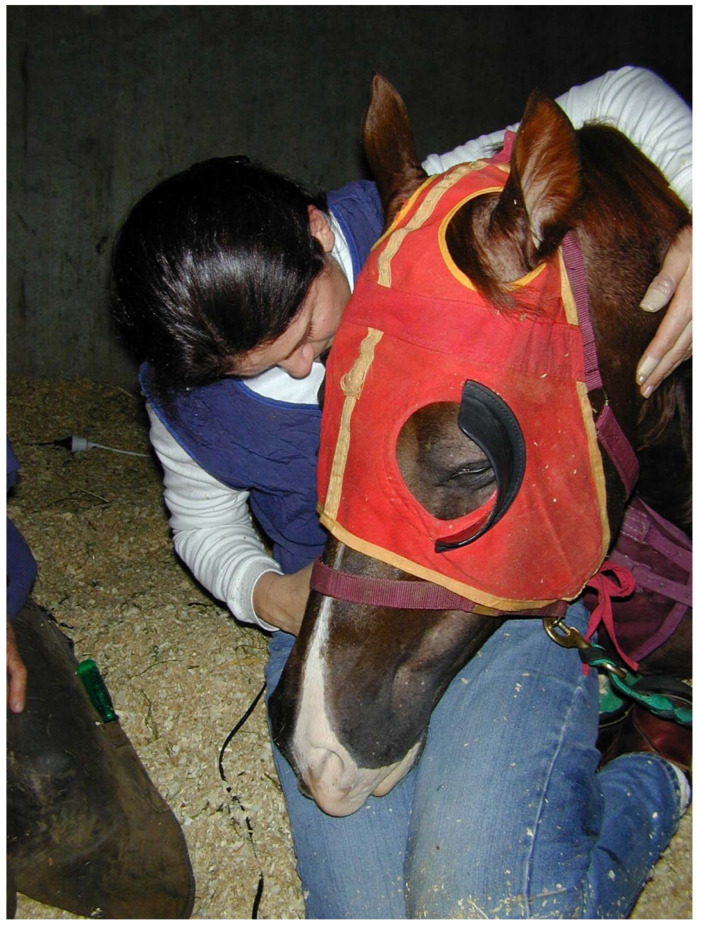
Pollitt, Christopher. *Interspecies trust: Francesca and Cambridge.* 2002. Digital image. Personal communication.

The practical and moral challenges of end-of-life decision making regarding horses belong to a different research paper; but the public exposure generated by media representations concerning Cambridge, were not anchored in a recognition of human limitations and did not accord the animal subject any agency in whether he should live or die. Rather, Cambridge became an object of wild speculation mediated by gendered, racialised, and classed discourses that were based on a reinscription of the human/animal divide, which feminists have long argued is structurally interrelated with other hierarchical dualisms (man versus woman, reason versus emotion, mind versus body, West versus non-West, and so on). In contrast, the autoethnographic narrative provided here aims to accentuate the need to attend to situated knowledges, among scientific and tacit knowledges, in order to ‘become *with*’ a horse in a relationship based on humility, curiosity, mutual vulnerability and trust. These different types of knowledges are central to forging a more nuanced ethics of animal welfare organised politically on behalf of animals and motivated by an intersectional ethics of care and responsibility. And whilst it is acknowledged that instrumental valuing—the use of an animal as a means toward an end that is defined as valuable by humans [56] (p. 142)—allows for, amongst other things, veterinary clinical practice, Cambridge’s demonstrations of feral resistance in his early days, and his insight into instinctual behaviour which enabled him to adapt to his personal circumstances during treatment, implores a more intrinsically based value system which recognises and enables a greater agency when humans engage with horses intersubjectively. Within the feminist care tradition in animal ethics this attention and acknowledgment of feeling and relationality should be considered alongside rights-based reliance on reason and abstract judgment and as such, becomes a “relational culture of caring and attentive love” [69] (p. 375): in other words, a radically different kind of ethics (Figure 3).

Words will never adequately describe the special relationship I shared with Cambridge, but Donna Haraway’s come close:

*We are training each other in acts of communication we barely understand. …. We make each other up, in the flesh. Significantly other to each other, in specific difference, we signify in the flesh a nasty developmental infection called love. This love is an historical aberration and a naturalcultural legacy*

[70] (p. 3).

Once the decision to euthanise Cambridge was made, and I’d re-bandaged his feet for the final time, I was left alone with him, not to face my grief or say goodbye, but just to *be*, tears held back for many months now, flowing as I buried my face against the silken skin of his neck.

## 8. Conclusions

Nonhuman animals are amongst the most vulnerable beings on the planet, yet dominant approaches to animal ethics, despite providing normative guidance about how humans ought to treat other animals, have arguably failed to provide the transformative change that early advocates, whether of the rights-based or feminist persuasions, had hoped for. Likewise, the thoroughbred racing and equestrian industries are frequently challenged by the public on welfare and ethics issues, which are routinely debated and mediated by popular forms of journalism and online commentary: animal stories sell. Understanding how popular culture produces and consumes animals as either beautiful or ugly, lovable, or unlovable, killable or unkillable, grievable or ungrievable has become critical as the field of animal studies searches for modes of analysis that might make sense of the paradoxical mix of care, sentiment, indifference and violence that has characterised human and animal relationships, past and present. Meanwhile, there are currently few reliable models for assessing stakeholder engagement on publicly mediated issues related to equine welfare, including end of life decision-making about elite sports horses, who as charismatic domestic megafauna, occupy a prominent place in human cultural and social imaginaries. Within these contexts, Cambridge’s story is offered as a case study to provoke thought about how the animal ethics promulgated by both welfare organisations and feminism alike might be further developed by paying greater attention to the moral obligations consequent upon the recognition of animal agency, and shared interspecies vulnerability, relationality, care and dependency. 

The embodied interspecies knowledge that informs the training and care of equines, as the paper has suggested, is always historically situated within permeable, dynamic worlds of self and other that are fluid, contextual, and always in relation. Feminist perspectives on moral reasoning about animals privilege contextual sensitivity and attention to the particular, rather than deferring to generalised moral rules or abstract reasoning. This foregrounding of the particular, extends to the category of ‘the animal’ itself, which in the singular prefaced by the indefinite article foregrounds the abstracted nature of a concept that allows humans to characterise members of particular nonhuman species as biological and transcultural constants. The use of thick description in the vignettes provided in the paper aims to narrativise and ‘bring to life’ time and context-sensitive interactions between a very particular horse and his carers as a praxis of an ethical position that includes relationships and emotions as critical to how people and animals function in real world situations. The paper has also used autoethnography to make visible structural features and institutional forces—whether cultural, economic, political, or social—that impact and shape the context in which moral choices about animals are made. By contrast, the simplifying, often abstracted and clichéd media representations of Cambridge’s incarceration at WPEC that were disseminated by television, radio and newspaper outlets and were in turn, sourced from unmoderated internet sites, belied the complexity that characterised the horse’s relationship with his significant others. Cambridge’s story also advises that we need to emotionally attend to what animals try to tell us about the kind of care they wish to receive, while also striving to care for animals in a way that creates space for their agency to emerge, even when that agency involves co-relational pain and loss. Such attentiveness is posited as a kind of caring moral perception that contains both emotional and cognitive dimensions, and as such, is susceptible to change and correction.

## Data Availability

The original contributions presented in the study are included in the article, further inquiries can be directed to the corresponding authors.
